# Nomograms established for predicting microvascular invasion and early recurrence in patients with small hepatocellular carcinoma

**DOI:** 10.1186/s12885-024-12655-2

**Published:** 2024-08-01

**Authors:** Xi Wang, Xinqun Chai, Ji Zhang, Ruiya Tang, Qinjunjie Chen

**Affiliations:** Department of Hepatological Surgery, Tongji Medical College, Union Hospital, Huazhong University of Science and Technology, Wuhan, China

**Keywords:** Small hepatocellular carcinoma, Microvascular invasion, Early recurrence, Prognosis, Nomogram

## Abstract

**Background:**

In this study, we aimed to establish nomograms to predict the microvascular invasion (MVI) and early recurrence in patients with small hepatocellular carcinoma (SHCC), thereby guiding individualized treatment strategies for prognosis improvement.

**Methods:**

This study retrospectively analyzed 326 SHCC patients who underwent radical resection at Wuhan Union Hospital between April 2017 and January 2022. They were randomly divided into a training set and a validation set at a 7:3 ratio. The preoperative nomogram for MVI was constructed based on univariate and multivariate logistic regression analysis, and the prognostic nomogram for early recurrence was constructed based on univariate and multivariate Cox regression analysis. We used the receiver operating characteristic (ROC) curves, area under the curves (AUCs), and calibration curves to estimate the predictive accuracy and discriminability of nomograms. Decision curve analysis (DCA) and Kaplan-Meier survival curves were employed to further confirm the clinical effectiveness of nomograms.

**Results:**

The AUCs of the preoperative nomogram for MVI on the training set and validation set were 0.749 (95%CI: 0.684–0.813) and 0.856 (95%CI: 0.805–0.906), respectively. For the prognostic nomogram, the AUCs of 1-year and 2-year RFS respectively reached 0.839 (95%CI: 0.775–0.903) and 0.856 (95%CI: 0.806–0.905) in the training set, and 0.808 (95%CI: 0.719–0.896) and 0.874 (95%CI: 0.804–0.943) in the validation set. Subsequent calibration curves, DCA analysis and Kaplan-Meier survival curves demonstrated the high accuracy and efficacy of the nomograms for clinical application.

**Conclusions:**

The nomograms we constructed could effectively predict MVI and early recurrence in SHCC patients, providing a basis for clinical decision-making.

## Introduction

Hepatocellular carcinoma (HCC) is a common malignant tumor of the digestive system. The latest data from GLOBOCAN showed that its incidence and mortality rates ranked 6th and 3rd among all malignant tumors [1]. In the Barcelona Clinic Liver Cancer (BCLC) system, potential radical treatments recommended for patients with early-stage HCC include radical resection, radiofrequency ablation (RFA) and liver transplantation [2]. In the majority of cases, surgical resection remains the first choice for HCC [3], but the 5-year postoperative recurrence rate could be as high as 70%, with most patients experiencing early recurrence within 2 years after surgery, which is inevitable even in small HCC (SHCC) (tumor diameter ≤ 3 cm) [4–6].

Microvascular invasion (MVI) is a major risk factor for recurrence of HCC, and preoperative detection of MVI is of great significance in the choice of diagnostic, therapeutic options, and prognosis [7]. However, MVI could only be diagnosed by postoperative pathology with a certain lag [8]. Some studies have shown that AFP level, inflammatory indexes, and gadoxetic acid–enhanced magnetic resonance imaging (EOB-MRI) features (tumor diameter and tumor margin) have a close relationship with MVI [9–11]. Nevertheless, previous studies have mainly focused on HCC, and few have comprehensively evaluated the relevant characteristics of MVI in SHCC patients, and proposed a preoperative prediction model for MVI with a good predictive performance.

For now, a lot of staging systems for HCC have been developed, such as the BCLC system, TNM system, Hong Kong Liver Cancer (HKLC) system and Japan Integrated Staging (JIS) score [12–15], which plays an important role in preoperative evaluation and postoperative treatment. However, none of them focus on SHCC early recurrence accurately [16]. Given the high early recurrence rate of HCC, accurate assessment of early recurrence in SHCC is essential for individualized treatment strategies [17]. Moreover, numerous studies have shown that SHCC patients with a high risk of recurrence required postoperative adjuvant therapy and careful follow-up [18, 19]. Recurrence-free survival (RFS) is significantly prolonged in HCC patients after receiving appropriate postoperative adjuvant therapy, especially in SHCC [20–22]. Therefore, the development of an appropriate SHCC early recurrence risk system is urgent.

Therefore, we retrospectively analyzed the clinical data of 326 SHCC patients in our hospital, and established and validated two nomograms for MVI and early recurrence in SHCC. The innovation of this study is to non-invasively predict the preoperative probability of MVI and early postoperative recurrence in SHCC patients, providing more accurate guidance for the intervention and treatment of SHCC patients.

## Methods

### Study design and study population

**Fig. 1:**
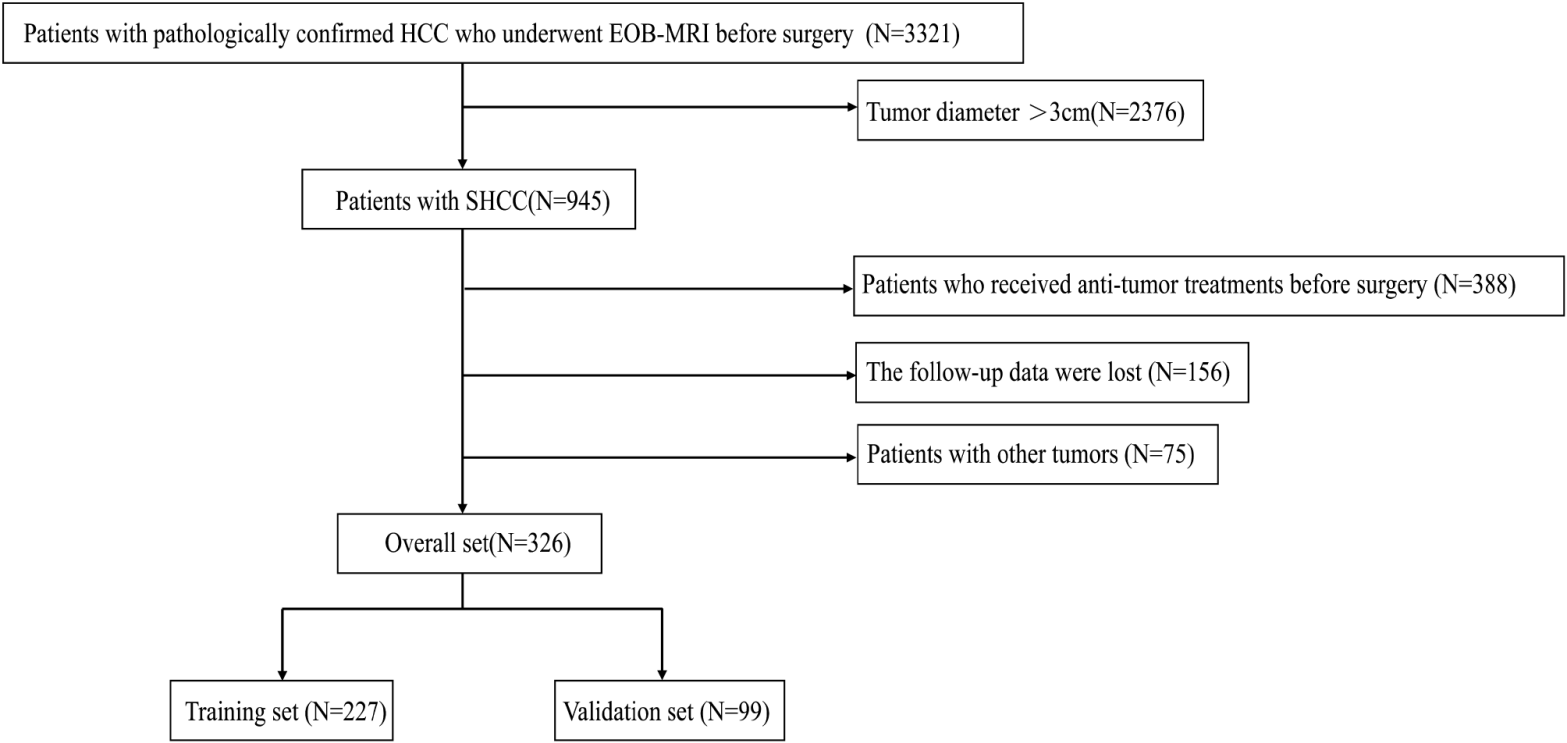
Flowchart of patient selection. HCC, hepatocellular carcinoma; EOB-MRI, gadoxetic acid–enhanced magnetic resonance imaging; SHCC, small hepatocellular carcinoma

This study retrospectively analyzed 326 SHCC patients who underwent radical resection at Wuhan Union Hospital between April 2017 and January 2022. This study was approved by Ethics Committee of Wuhan Union Hospital and did not require informed consent from participants (Ethics approval number: 2023 − 0586). According to the inclusion and exclusion criteria, 326 patients were enrolled in the study. Inclusion criteria included: (1) single tumor ≤ 3 cm in diameter or the sum of two tumors ≤ 3 cm in diameter; (2) patients underwent radical resection with definite pathological diagnosis; (3) received EOB-MRI preoperatively; (4) patients of clear mind and normal intelligence who could cooperate with the relevant examinations. Exclusion criteria included: (1) patients who underwent anti-tumor treatments such as surgical resection, transcatheter arterial chemoembolization (TACE), local ablation, targeted immunotherapy and liver transplantation before surgery; (2) patients with clinical and follow-up data missing; (3) pregnant and lactating female; (4) patients with surgical margin positive; (5) patients with other malignant tumors. For analysis, all 326 patients were randomly divided into a training set and a validation set at a 7:3 ratio (Fig. 1). The nomograms were established using the training set and its accuracy was validated using the validation set.

### Collection of data and definition of variables

**Fig. 2:**
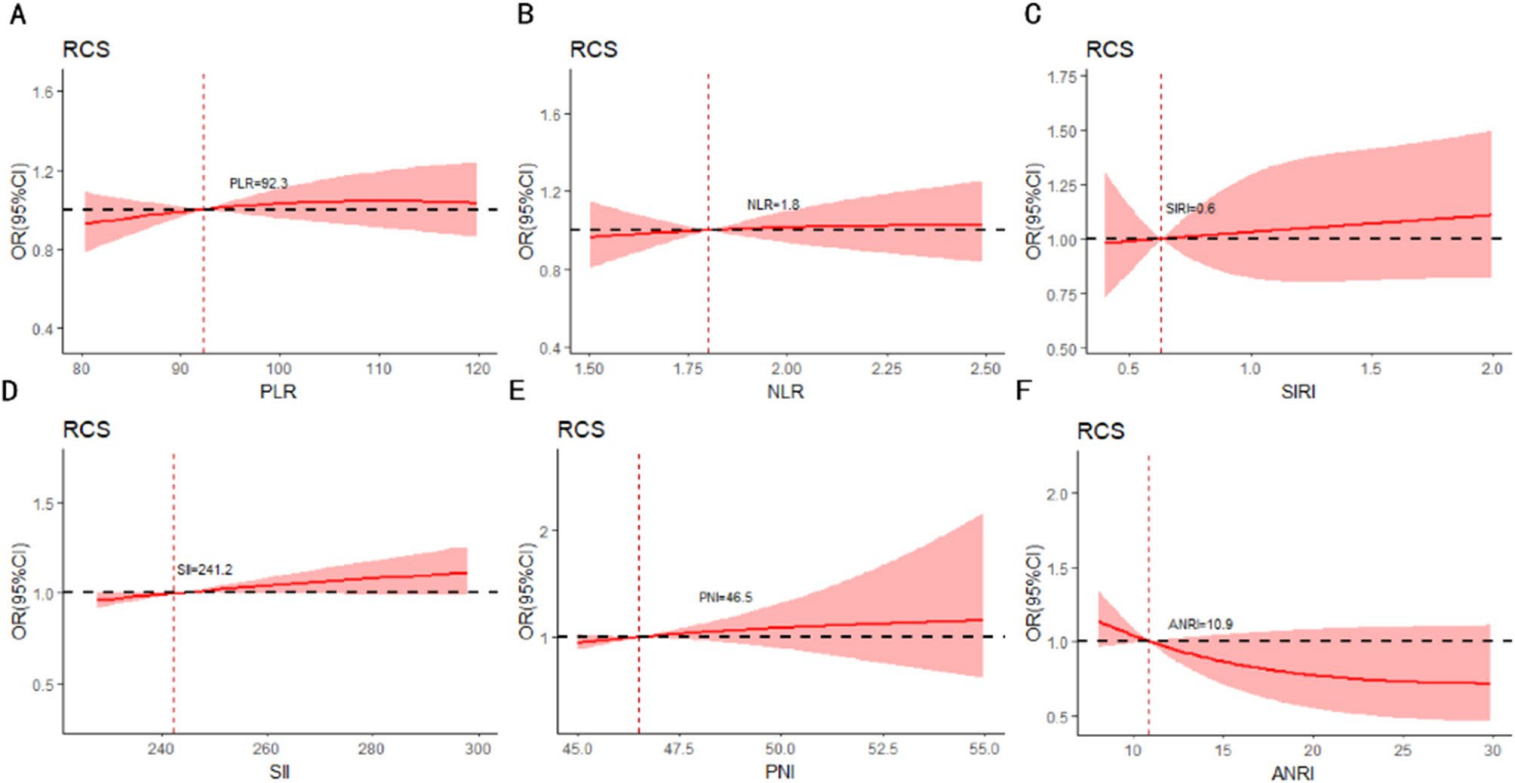
The cut-off value of the inflammatory biomarkers for predicting SHCC with MVI. PLR, platelet-to-lymphocyte ratio (**A**); NLR, neutrophil-to-lymphocyte ratio (**B**); SIRI, systemic inflammation response index (**C**); SII, systemic immune-inflammation index (**D**); PNI, prognostic nutritional index (**E**); ANRI, aspartate aminotransferase to neutrophil ratio index (**F**)

Baseline data collected included patient characteristics, laboratory index, inflammatory biomarkers, radiomics features, histopathologic characteristic, surgical information and follow-up data. Patient characteristics included age, sex, body mass index (BMI), etiology, cirrhosis, Child-Pugh grade, ALBI stage and BCLC grade. Laboratory index involved aspartate aminotransferase (AST), alanine aminotransferase (ALT), total bilirubin (TBIL), albumin (ALB), platelet (PLT), prothrombin time (PT) and alpha-fetoprotein (AFP). Inflammatory biomarkers included platelet-to-lymphocyte ratio (PLR), neutrophil-to-lymphocyte ratio (NLR), systemic inflammation response index (SIRI), systemic immune-inflammation index (SII), aspartate aminotransferase to neutrophil ratio index (ANRI), prognostic nutritional index (PNI). Radiomics features incorporated tumor diameter, tumor number, tumor location and tumor margin. Histopathologic characteristic involved MVI and Edmondson-Steiner grade. MVI was defined as a tumor cell nest that was only visible under the microscope in the tumor capsule blood vessels of the portal vein, hepatic vein, and endothelial lining. The “7-point” baseline sampling method was used for evaluation [23]. The three-tiered MVI grading system (MVI-TTG) classified specimens as M0 (no MVI detected), M1 (≤ 5 MVIs, all occurring in adjacent liver tissue ≤ 1 cm away from the main tumor), and M2 (> 5 MVIs or any MVI occurring in adjacent liver tissue ≤ 1 cm away from the main tumor) [24]. Surgical information included surgical methods and surgical margin. Among them, surgical methods included AR (anatomic resection) and NAR (non-anatomic resection). All laboratory index and radiomics features were obtained up to 1 week before surgery. The inflammatory biomarkers were calculated by the following formula: SIRI = (neutrophil × monocyte)/lymphocyte; SII = PLT × (neutrophil/lymphocyte); ANRI = AST/neutrophil; PNI = ALB + 5 × lymphocyte. The ALBI score was computed by the formula: ALBI = (log10(TBIL) × 0.66 + ALB× (− 0.085)). The cut-off value of the inflammatory biomarkers for predicting SHCC with MVI in our study were set by plotting the restricted cubic splines (PLR = 92.3; NLR = 1.8; SIRI = 0.6; SII = 241.2; PNI = 46.5;  ANRI = 10.9), as shown in Fig. 2. Besides, AST, ALT, TBIL, ALB, PLT and PT cut-off value in our study were used as the upper limit of normal values for serologic tests in our institution.

### Follow-up data

All patients underwent radical resection, defined as the complete resection of tumor tissue with negative surgical margin. After surgery, all patients were followed up monthly for the first three months, then every two months for the first year, and every three months thereafter. Laboratory index (including serum AFP level and blood tests) and imaging examinations (contrast-enhanced ultrasound, computed tomography or EOB-MRI) were conducted in follow-up examinations. Early recurrence was defined as the recurrence of HCC within 2 years after radical resection. HCC preoperative and recurrence diagnosis were both based on the criteria stipulated in the 2022 Standard for diagnosis and treatment of primary liver cancer in China [25]. The main end-point of our study was RFS, which was defined as the time from the date of radical resection to the date of tumor recurrence or the last follow-up without early recurrence within 2 years.

### Statistical analysis

Continuous variables were expressed as mean ± standard deviation and compared by Student’s t test. Categorical variables were expressed as frequency and percentage, and the chi-square test or Fisher’s exact test was used for comparison. LASSO regression analysis was used for data dimensionality reduction and element selection. In the training set, independent risk factors for MVI were identified by univariable and multivariable logistic analysis, and independent prognostic factors of SHCC early recurrence were identified by univariable and multivariable Cox proportional hazard regression analysis. Subsequently, we established two nomograms to predict the risk of MVI and RFS in SHCC. ROC curves were plotted to assess nomogram’s differentiation and predictive efficacy in terms of area under the curve (AUC). Calibration curves were plotted to assess the agreement of nomograms, and decision curve analysis (DCA) was plotted to assess the clinical application value of nomograms by demonstrating net benefit for each risk threshold probability. We compared the ROC curves, the AUCs, the calibration curves, and the DCA results between the training and validation set to verify the stability of the nomogram. Finally, patients in the training set and validation set were assigned to either the high-risk group or the low-risk group based on the median risk score of the prognostic nomogram. RFS curves were calculated using the Kaplan–Meier method and compared with the Log-rank test. All statistical analyses were conducted using SPSS (version 26.0) and R software (version 4.3.1). Two-tailed *P* value < 0.05 was considered as a measure of statistical significance.

## Results

### Baseline clinical characteristics

A total of 326 patients with SHCC receiving radical resection were included in our study. Of these, 227 patients were assigned to the training set and 99 patients to the validation set. Patients baseline clinical characteristics were summarized in Table 1. There were no differences in clinical, radiologic, histopathologic characteristics or follow-up information between the training and validation sets (all *P* > 0.05). The median RFS was 19.2 months (95% CI: 12.3–26.1) for the training set and 18.3 months (95% CI:11.5–25.1) for the validation set (*P* = 0.249).

**Table 1:** Patients baseline clinical characteristics

Variable	Overall set (*n* = 326)	Training set (*n* = 227)	Validation set (*n* = 99)	*P* value
**Patient characteristic**				
Age, years	56.1 ± 10.6	55.5 ± 10.4	57.4 ± 10.8	0.147
Sex (Male/Female)	277/49	195/32	82/17	0.475
BMI	23.6 ± 3.1	23.5 ± 3.1	24.0 ± 2.9	0.226
Etiology				0.098
HBV	272 (83.4)	194 (85.5)	78 (78.8)	
HCV	21 (6.4)	12 (5.3)	9 (9.1)	
HBV and HCV	4 (1.2)	4 (1.8)	0 (0.0)	
Others	29 (8.9)	17 (7.5)	12 (12.1)	
Cirrhosis (Yes)	282 (86.5)	196 (86.3)	86 (86.6)	0.898
Child-Pugh grade (A/B)	277/49	193/34	84/15	0.968
ALBI grade				0.260
1	160 (49.1)	118 (52.0)	42 (42.4)	
2	162 (49.7)	106(46.7)	56 (56.6)	
3	4 (1.2)	3 (1.3)	1 (1.0)	
BCLC grade (0/A)	74/252	53/174	21/78	0.672
**Laboratory index**				
AST ≥ 35 IU/L	100 (30.7)	70 (30.8)	30 (30.3)	0.923
ALT ≥ 40 IU/L	83 (25.5)	55 (24.2)	28 (28.3)	0.440
TBIL ≥ 19 µmol/L	87 (26.7)	63 (27.8)	24 (24.2)	0.510
ALB < 35 g/L	56 (17.2)	38 (16.7)	18 (18.2)	0.751
PLT < 100 × 10^9^/L	84 (25.8)	58 (25.6)	26 (26.3)	0.892
PT ≥ 14.2 s	56 (17.2)	67 (29.5)	29 (29.3)	0.968
AFP ≥ 200ng/mL	81 (24.8)	63 (27.8)	18 (18.2)	0.066
**Inflammatory Biomarkers**				
PLR ≥ 92.3	160 (49.1)	113 (49.8)	47 (47.5)	0.702
NLR ≥ 1.8	43 (13.2)	29 (12.8)	14 (14.1)	0.737
SIRI ≥ 0.6	175 (53.7)	121 (53.3)	54 (54.5)	0.836
SII ≥ 241.2	156 (47.9)	111 (48.9)	45 (45.5)	0.567
ANRI ≥ 10.9	180 (55.2)	123 (54.2)	57 (57.6)	0.571
PNI ≥ 46.5	159 (48.8)	107 (47.1)	52 (52.5)	0.371
**Radiomics features**				
Tumor diameter, cm	2.3 ± 0.6	2.3 ± 0.6	2.3 ± 0.6	0.982
Tumor number (1/2)	317/9	221/6	96/3	0.844
Tumor location				0.546
Left lobe	83 (25.5)	61 (26.9)	22 (22.2)	
Right lobe	236 (72.4)	162 (71.4)	74 (74.7)	
Caudal lobe	7 (2.1)	4 (1.8)	3 (3.0)	
Tumor margin (Non-smooth)	104 (31.9)	75 (33.0)	29 (29.3)	0.505
**Histopathologic characteristic**				
MVI				0.649
M0	202 (62.0)	137 (60.4)	65 (65.7)	
M1	100 (30.7)	73 (32.2)	27 (27.3)	
M2	24 (7.4)	17 (7.5)	7 (7.1)	
Edmondson-Steiner				0.657
I-II	174 (53.4)	123 (54.2)	51 (51.5)	
III-IV	152 (46.6)	104 (45.8)	48 (48.5)	
**Surgical information**				
Surgical methods				0.162
AR	276 (84.7)	188 (82.8)	88 (88.9)	
NAR	50 (15.3)	39 (17.2)	11 (11.1)	
Surgical margin, cm				0.772
< 1	212 (65.0)	145 (63.9)	67 (67.7)	
1–2	63 (19.3)	46 (20.3)	17 (17.2)	
≥ 2	51 (15.6)	36 (15.9)	15 (15.2)	
**Recurrence**	135 (41.4)	93 (41.0)	42 (42.4)	0.806
RFS				0.302
1-year rate, %	72.7	74.9	67.7	
2-year rate, %	56.7	59.0	51.5	
Median, months	18.9 ± 6.9	19.2 ± 6.9	18.3 ± 6.8	0.249

Data are expressed as n (%) or median (interquartile range)

BMI, body mass index; HBV, hepatitis B virus; HCV, hepatitis C virus; AST, aspartate aminotransferase; ALT, alanine aminotransferase; TBIL, total bilirubin; ALB, albumin; PLT, platelet; PT, prothrombin time; AFP, alpha-fetoprotein; PLR, platelet-to-lymphocyte ratio; NLR, neutrophil-to-lymphocyte ratio; SIRI, systemic inflammation response index; SII, systemic immune-inflammation index; ANRI, aspartate aminotransferase to neutrophil ratio index; PNI, prognostic nutritional index; ALBI, albumin-bilirubin; BCLC, Barcelona Clinic Liver Cancer; MVI, microvascular invasion; AR, anatomic resection; NAR, non-anatomic resection; RFS, recurrence-free survival

### Independent risk factors for MVI

**Fig. 3:**
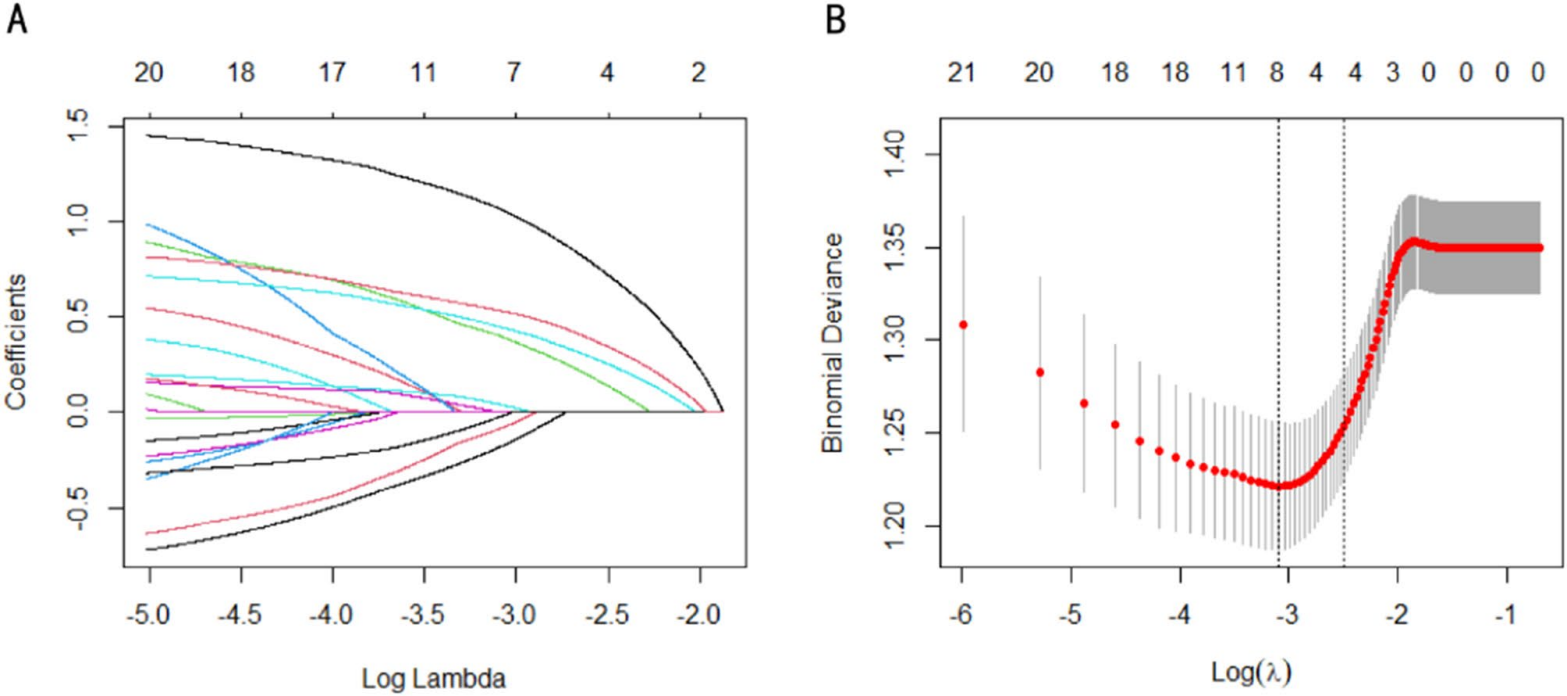
Lasso regression identifying the independent risk factors for MVI at training set. Lasso regression coefficients (**A**); Lasso regression cross-validation diagram (**B**)

Patient characteristics, laboratory index, inflammatory biomarkers and radiomics features in Table 1 were included in Lasso regression analysis for element selection (Fig. 3). Table 2 illustrated that univariable logistic analysis demonstrated that age ≥ 60 years, TBIL ≥ 19µmol/L, AFP ≥ 200ng/mL, NLR ≥ 1.8, PNI ≥ 46.5, larger tumor diameter, and tumor margin non-smooth were significantly associated with MVI (all *P* < 0.05). The multivariable logistic analysis showed that AFP ≥ 200ng/mL, NLR ≥ 1.8, PNI ≥ 46.5, larger tumor diameter, and tumor margin non-smooth were independent risk factors for MVI (all *P* < 0.05).

**Table 2:** Univariable and multivariable logistic regression of risk factor for SHCC with MVI

Variable	Univariate analysis			Multivariate analysis		
	OR	95%CI	*P* value	OR	95%CI	*P* value
Age ≥ 60 years	0.513	0.282–0.934	0.029	0.522	0.266–1.024	0.059
Sex (Male)	1.412	0.666–2.994	0.369			
BMI	0.971	0.890–1.058	0.496			
Etiology						
HBV	1.314	0.467–3.699	0.605			
HCV	0.367	0.060–2.252	0.279			
HBV and HCV	0.611	0.052–7.240	0.696			
Others	Ref.	Ref.	Ref.			
Cirrhosis (Yes)	0.896	0.415–1.933	0.779			
AST ≥ 35 IU/L	0.719	0.400-1.293	0.271			
ALT ≥ 40 IU/L	0.833	0.444–1.560	0.568			
TBIL ≥ 19 µmol/L	0.511	0.273–0.957	0.036	0.699	0.356–1.374	0.299
ALB < 35 g/L	0.991	0.486–2.022	0.981			
PLT < 100 × 10^9^/L	0.606	0.321–1.143	0.122			
PT ≥ 14.2 s	0.661	0.364–1.203	0.176			
AFP ≥ 200 ng/mL	2.065	1.145–3.724	0.016	2.723	1.287–5.762	0.009
PLR ≥ 92.3	1.705	0.996–2.917	0.052			
NLR ≥ 1.8	4.908	2.066–11.658	< 0.001	4.103	1.577–10.817	0.004
SIRI ≥ 0.6	1.349	0.789–2.305	0.208			
SII ≥ 241.2	1.557	0.912–2.660	0.105			
ANRI ≥ 10.9	0.877	0.515–1.496	0.631			
PNI ≥ 46.5	0.532	0.309–0.916	0.023	0.472	0.247–0.903	0.023
Child-Pugh grade (B)	1.242	0.595–2.594	0.564			
Tumor diameter, cm	2.726	1.677–4.429	< 0.001	2.467	1.439–4.231	0.001
Tumor number (2)	1.540	0.304–7.806	0.602			
Tumor location						
Left lobe	3.310	0.326–33.627	0.312			
Right lobe	1.629	0.166–16.018	0.676			
Caudal lobe	Ref.	Ref.	Ref.			
Tumor margin (Non-smooth)	3.567	2.000-6.361	< 0.001	2.735	1.432–5.442	0.002

#### Preoperative Nomogram for MVI Establishment and Validation

**Fig. 4:**
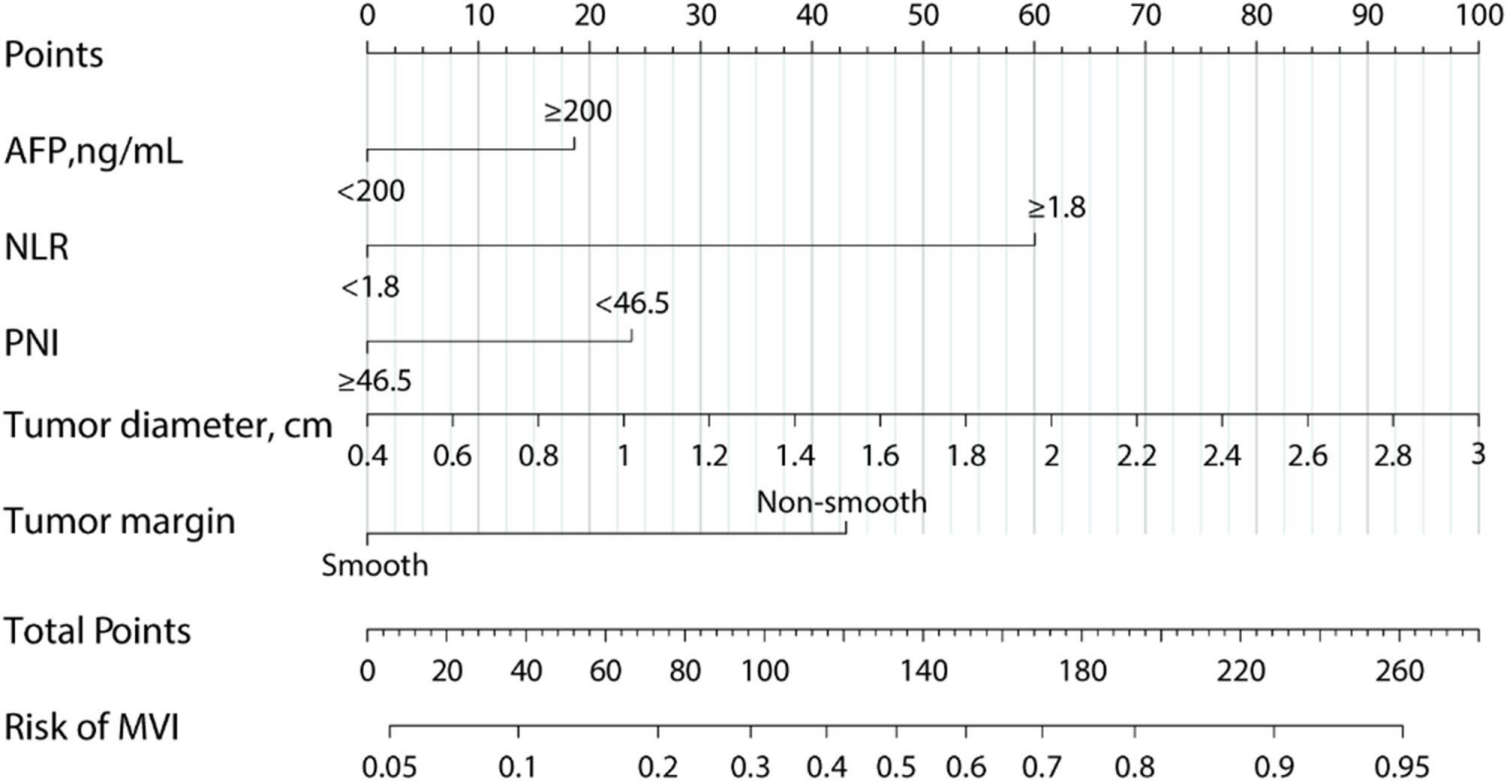
Nomogram to predict the risk of MVI in patients with SHCC.AFP, alpha-fetoprotein; NLR, neutrophil-to-lymphocyte ratio; PNI, prognostic nutritional index; MVI, microvascular invasion

**Fig. 5:**
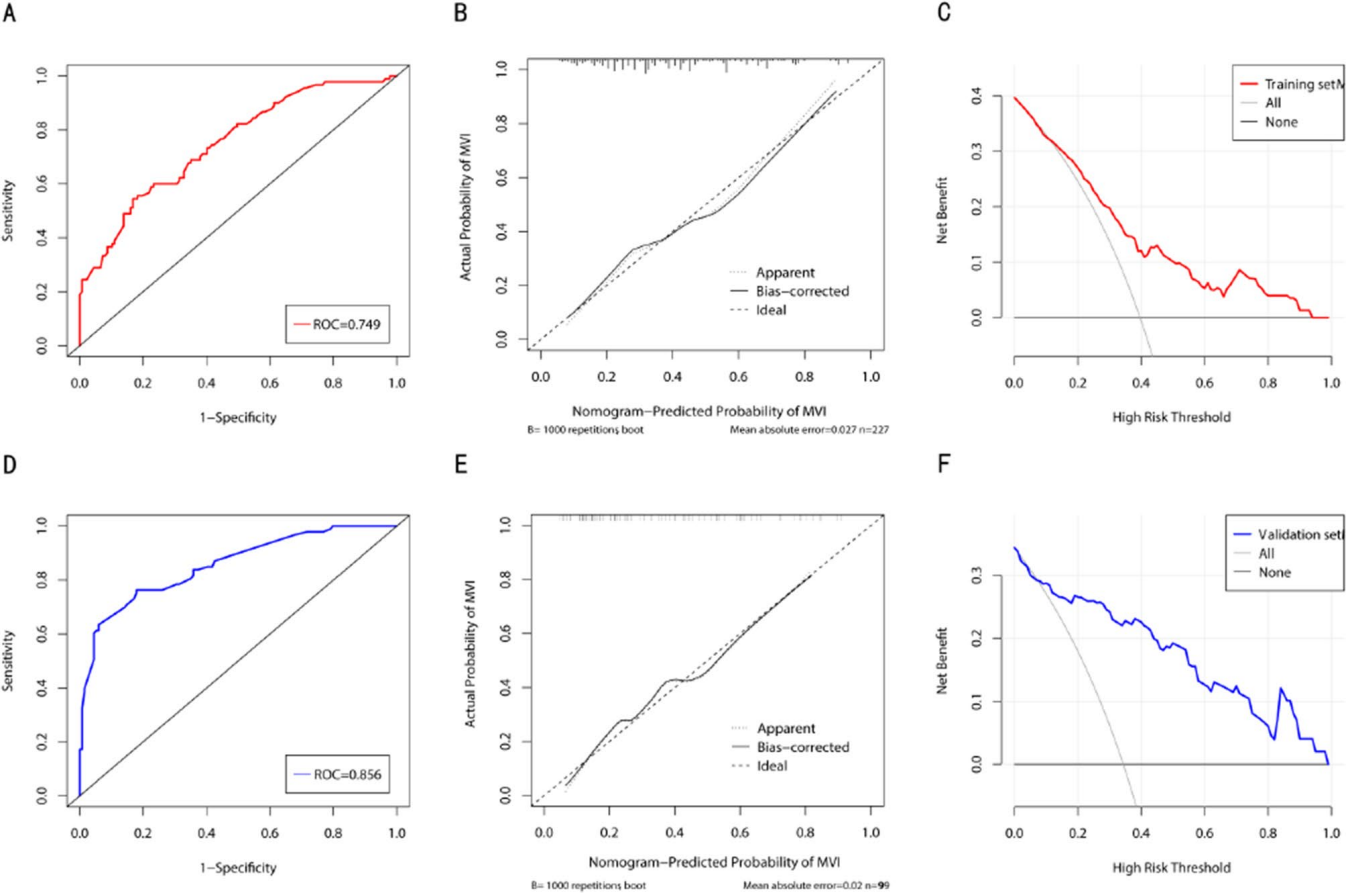
The ROC curves for predicting the risk of SHCC with MVI in the training set (**A**) and validation set (**D**). The calibration curves for predicting the risk of SHCC with MVI in the training set (**B**) and validation set (**E**). The DCA for the training set (**C**) and the validation set (**F**). ROC receiver operating characteristic; DCA decision curve analysis

Based on above 5 independent risk factors, a nomogram for predicting the risk of MVI in patients with SHCC was constructed (Fig. 4). In the training set, the nomogram achieved an AUC of 0.749 (95%CI: 0.684–0.813) (Fig. 5A). In the validation set, the nomogram had an AUC of 0.856 (95%CI: 0.805–0.906) (Fig. 5D). There was no statistically significant difference in the AUC between the training and validation set (*P* > 0.05), indicating that the prediction nomogram had a high discriminative ability. The calibration curve showed good agreement between the predicted and actual probabilities in the training set (Fig. 5B). In the validation set, the calibration curve was slightly less consistent with the actual probabilities, but they were close to each other, demonstrating that the nomogram had a good level of reproducibility and reliability (Fig. 5E). DCA of the nomogram revealed the nomogram had a higher net benefit than categorizing all patients as MVI across almost all threshold probabilities (Fig. 5C and F).

### Independent prognostic factors for SHCC

**Fig. 6:**
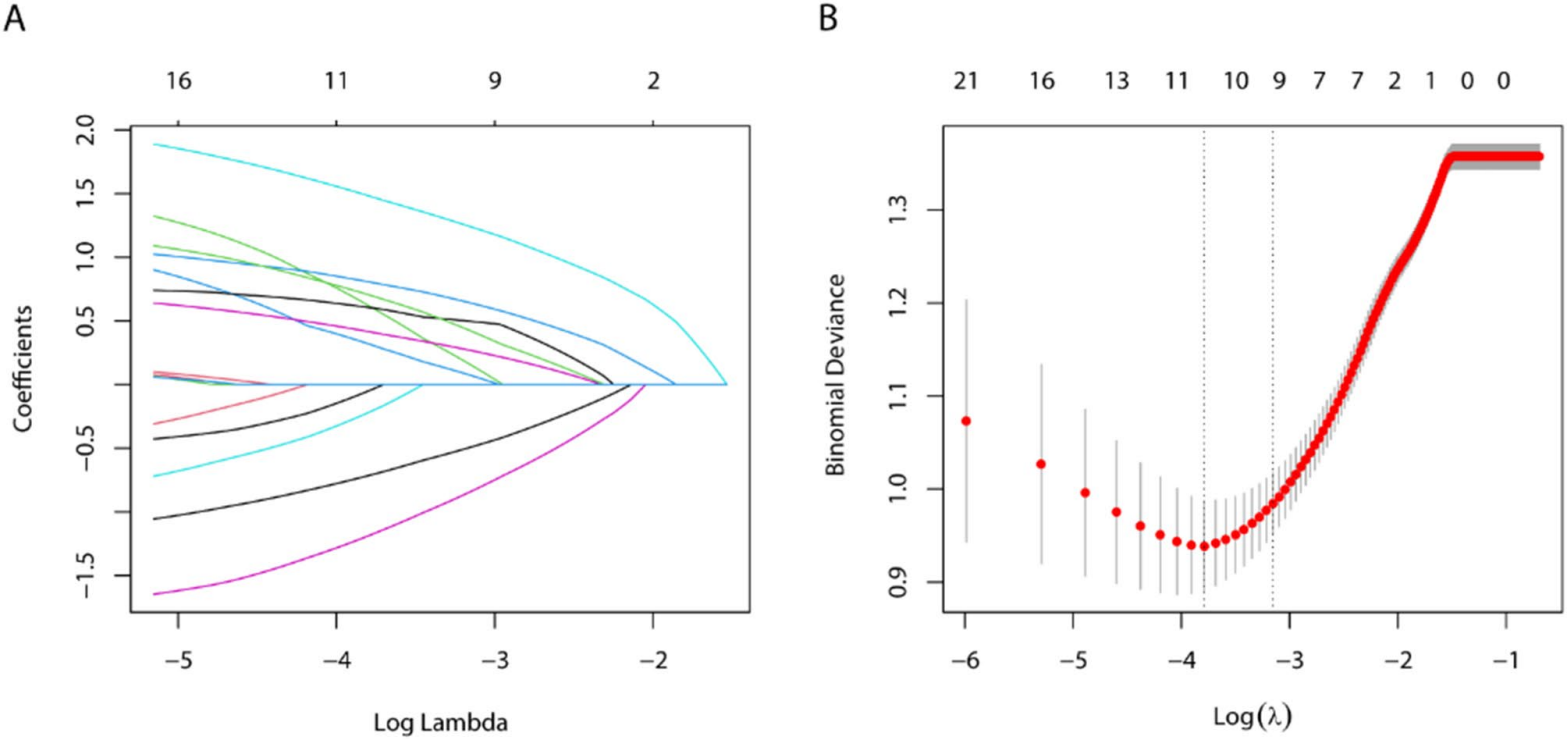
Lasso regression identifying the prognostic factors of RFS at training set. Lasso regression coefficients (**A**); Lasso regression cross-validation diagram (**B**)

Included patient characteristics, laboratory index, radiomics features, histopathologic characteristic and surgical information into the Lasso regression analysis (Fig. 6). The characteristics screened in the Lasso regression analysis were further revealed by univariable and multivariable Cox proportional hazard regression analysis. Our univariable Cox analysis revealed that ALB < 35 g/L, AFP ≥ 200 ng/mL, Child-Pugh B, BCLC A, larger tumor diameter, MVI, Edmondson-Steiner III-IV, NAR, and surgical margin < 1 cm were significantly associated with tumor recurrence (all *P* < 0.05). The multivariable Cox analysis showed that AFP ≥ 200 ng/mL, MVI as M2, Edmondson-Steiner III-IV, NAR, and surgical margin < 1 cm were independent prognostic factors of RFS in Table 3 (all *P* < 0.05).

**Table 3:** Univariable and multivariable Cox proportional hazard regression analysis for SHCC with recurrence

Variable	Univariate analysis			Multivariate analysis			
	HR	95%CI	*P* value	HR	95%CI	*P* value	
Age ≥ 60 years	0.936	0.601–1.458	0.769				
Sex (Male)	1.119	0.634–1.977	0.697				
Cirrhosis (Yes)	1.889	0.915–3.901	0.085				
Child-Pugh grade (B)	1.971	1.229–3.160	0.005	0.758	0.298–1.924	0.559	
ALBI grade							
1	Ref.	Ref.	Ref.				
2	1.127	0.745–1.703	0.571				
3	3.022	0.939–9.734	0.064				
BCLC grade (A)	2.977	1.544–5.740	0.001	2.317	0.922–5.821	0.074	
AST ≥ 35 IU/L	0.991	0.641–1.531	0.967				
ALT ≥ 40 IU/L	0.976	0.609–1.564	0.920				
TBIL ≥ 19 µmol/L	1.349	0.873–2.084	0.178				
ALB < 35 g/L	1.880	1.181–2.993	0.008	1.865	0.760–4.575	0.174	
PLT < 100 × 10^9^/L	0.985	0.615–1.578	0.915				
PT ≥ 14.2 s	1.265	0.825–1.941	0.282				
AFP ≥ 200 ng/mL	2.068	1.363–3.136	0.001	2.161	1.368–3.414	0.001	
Tumor diameter, cm	1.628	1.146–2.313	0.007	0.823	0.469–1.445	0.497	
Tumor number (2)	0.834	0.250–3.387	0.800				
Tumor margin (Non-smooth)	1.426	0.939–2.165	0.096				
MVI							
M0	Ref.	Ref.	Ref.				
M1	2.147	1.377–3.349	0.001	1.458	0.869–2.448	0.153	
M2	7.090	3.845–13.072	< 0.001	6.475	3.142–13.345	< 0.001	
Edmondson-Steiner (III-IV)	4.127	2.617–6.510	< 0.001	3.150	1.894–5.237	< 0.001	
Surgical methods (NAR)	2.881	1.845–4.499	< 0.001	2.022	1.250–3.272	0.004	
Surgical margin, cm							
< 1	Ref.	Ref.	Ref.				
1–2	0.457	0.253–0.826	0.009	0.267	0.140–0.509	< 0.001	
≥ 2	0.366	0.176–0.759	0.007	0.277	0.129–0.591	0.001	

### Prognostic nomogram establishment and validation

**Fig. 7:**
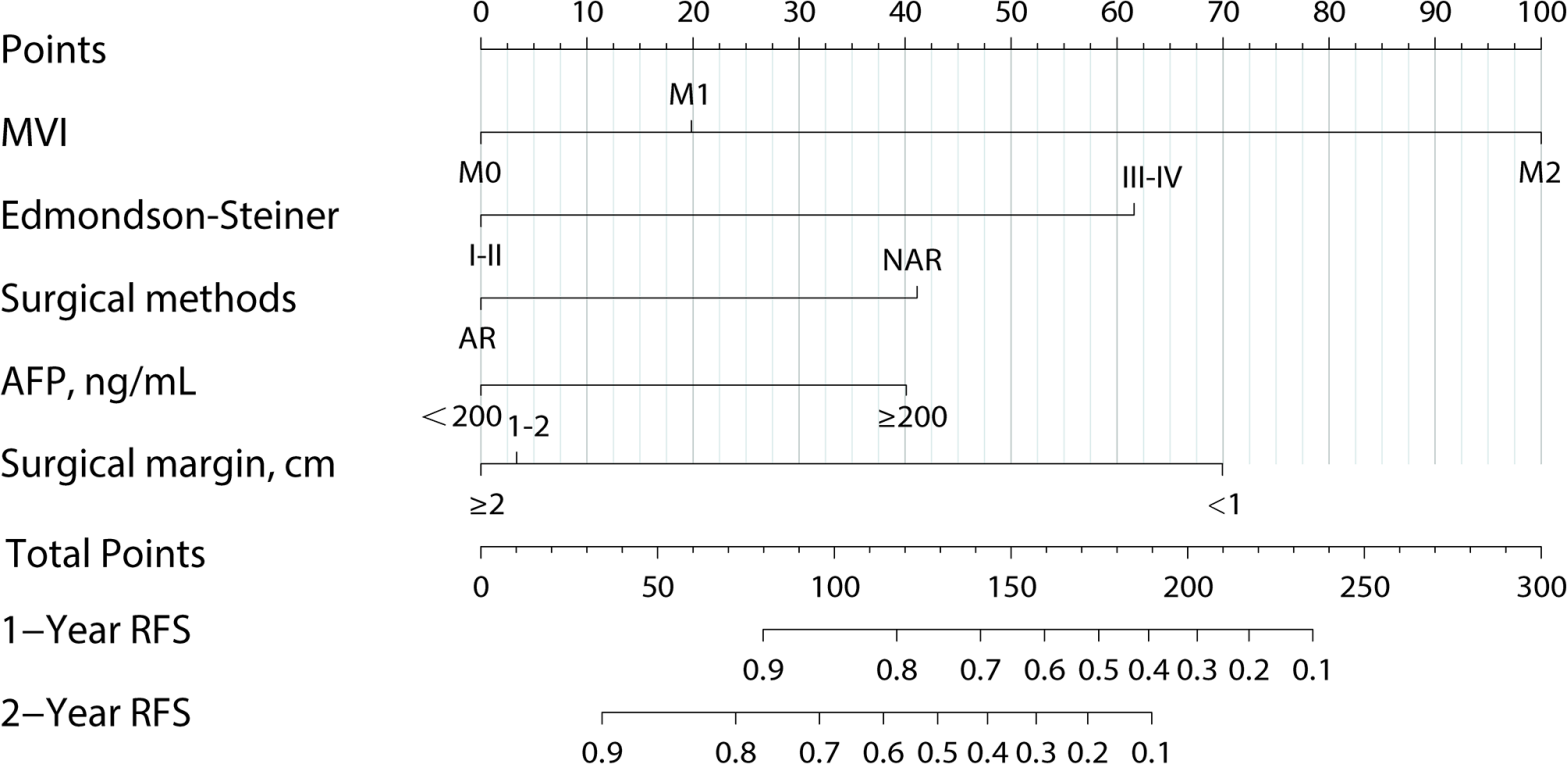
Nomogram for predicting the 1-year and 2-year recurrence in patients with SHCC

**Fig. 8:**
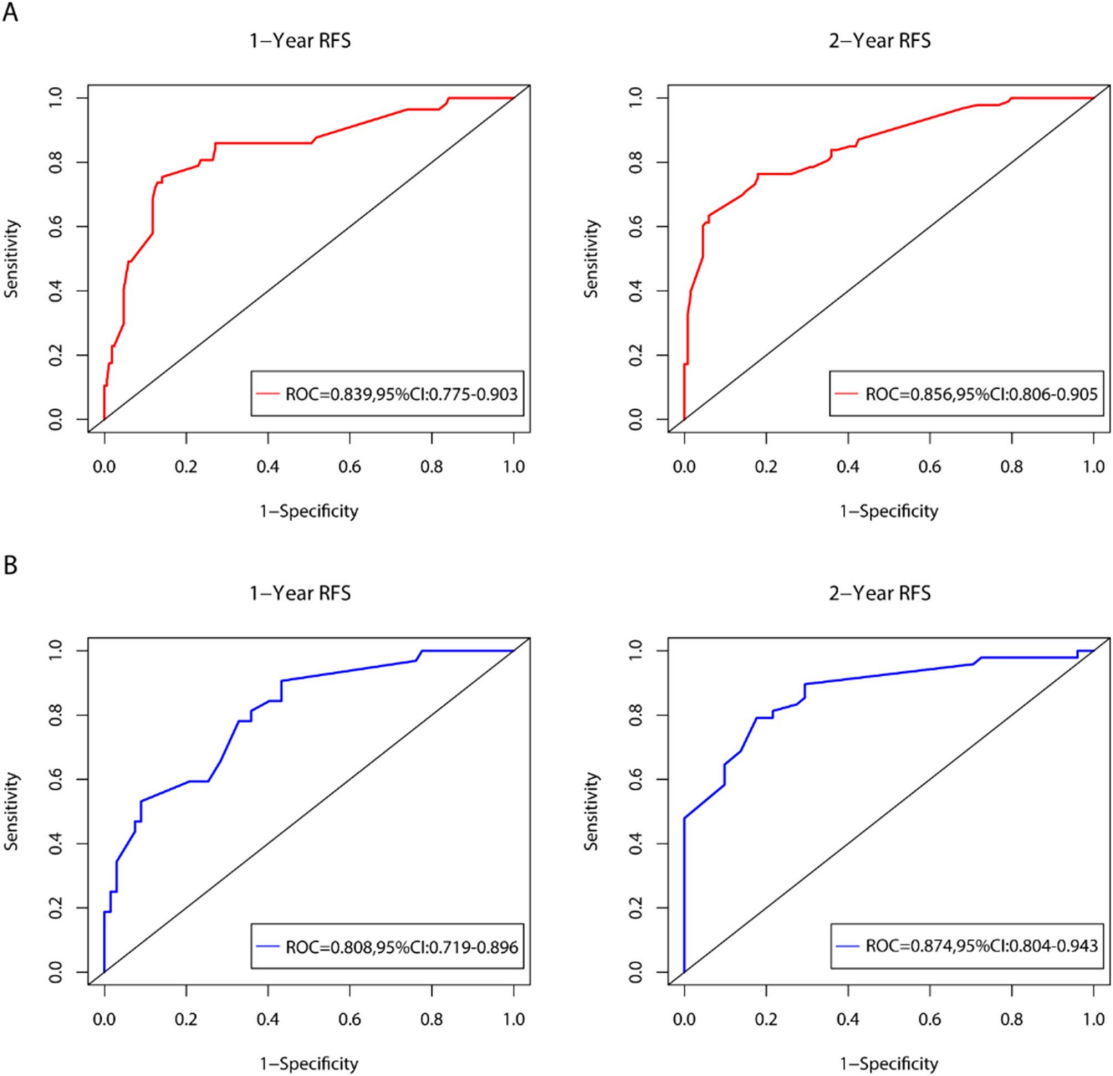
ROC curves of the ability of nomogram to predict 1-year and 2-year RFS in training set (**A**) and validation set (**B**)

**Fig. 9:**
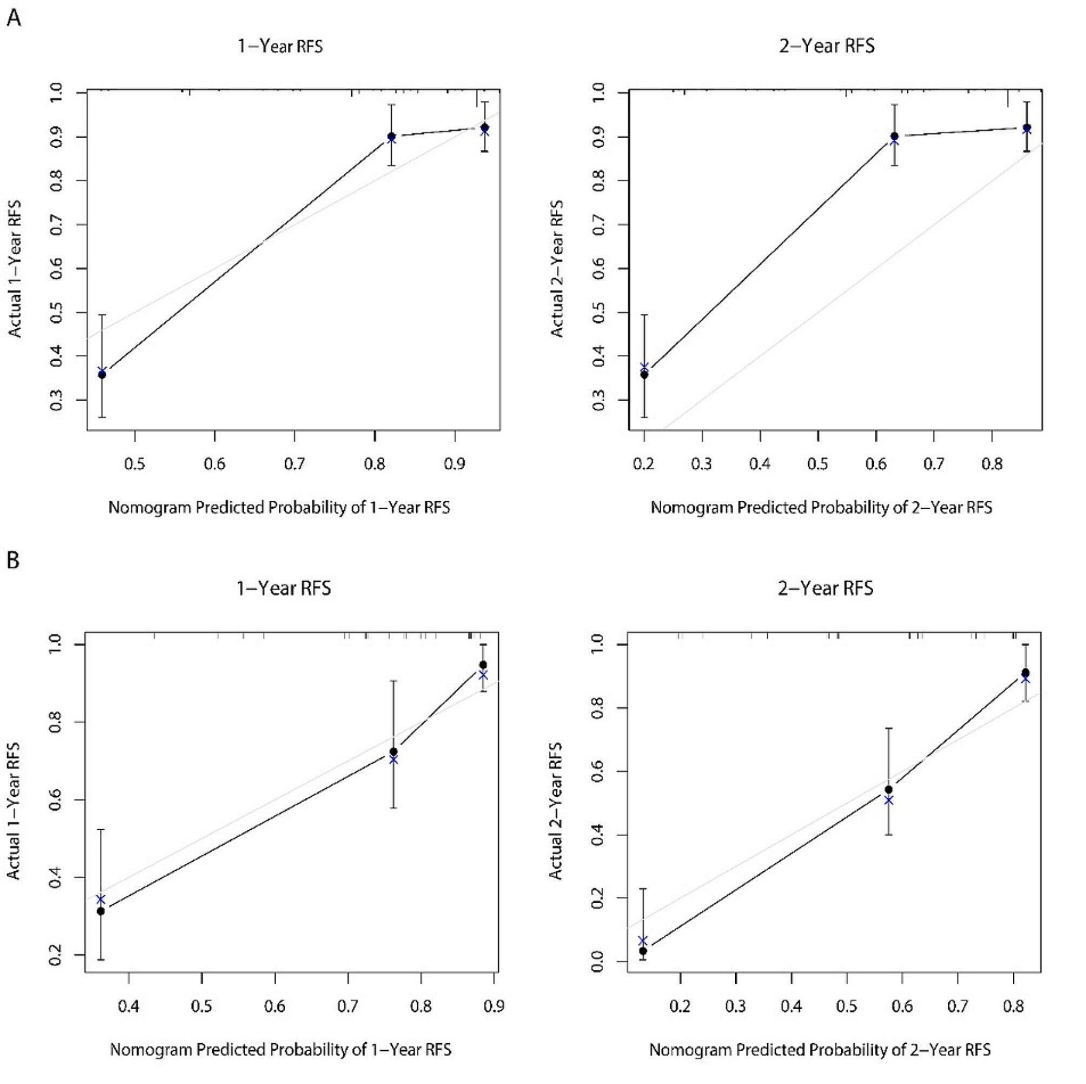
Calibration curves of the nomogram. Calibration curves of 1-year and 2-year RFS for SHCC patients in training set (**A**) and validation set (**B**)

**Fig. 10:**
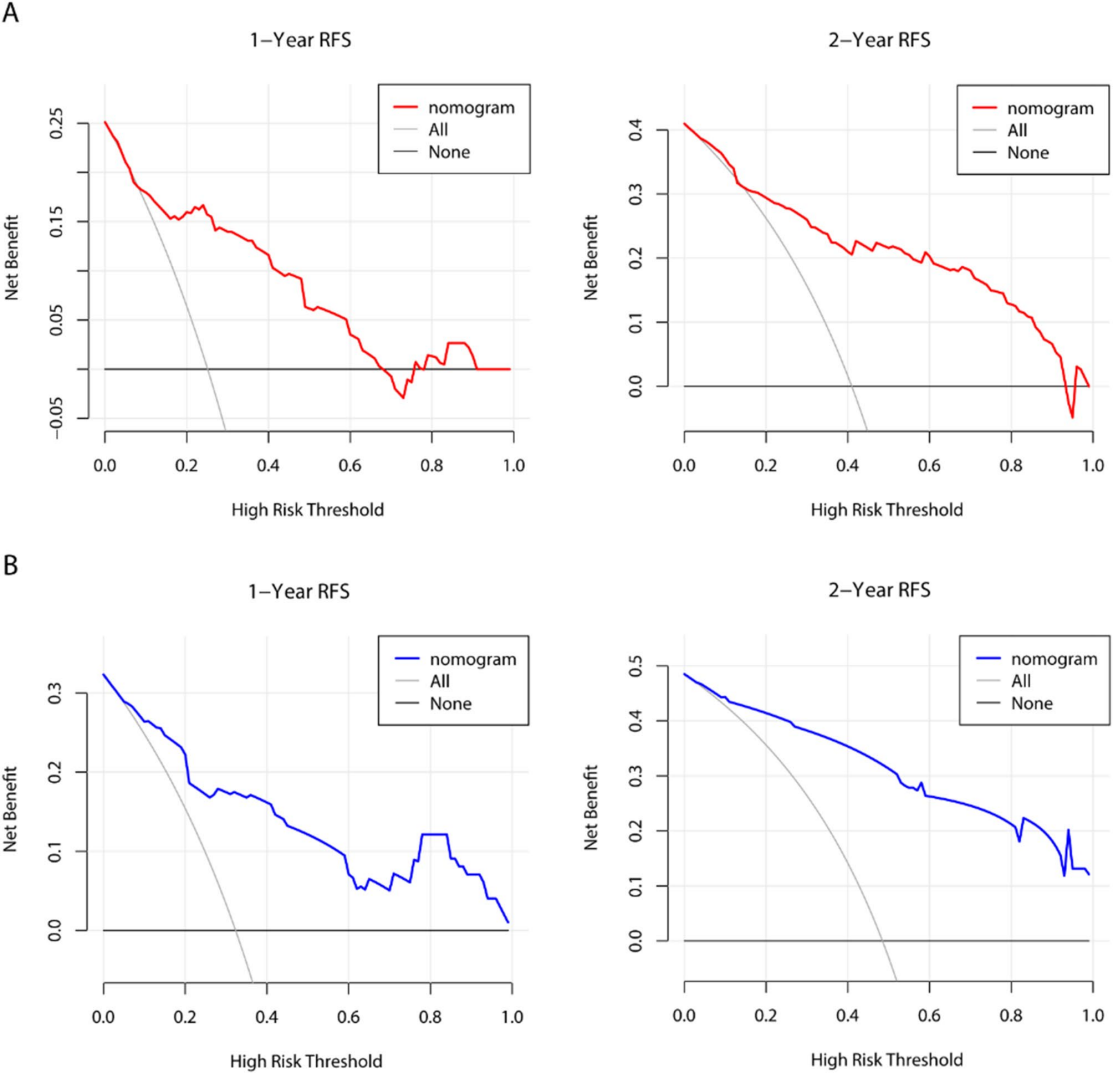
DCA of the nomogram. DCA of 1-year and 2-year RFS for SHCC patients in training set (**A**) and validation set (**B**)

**Fig. 11:**
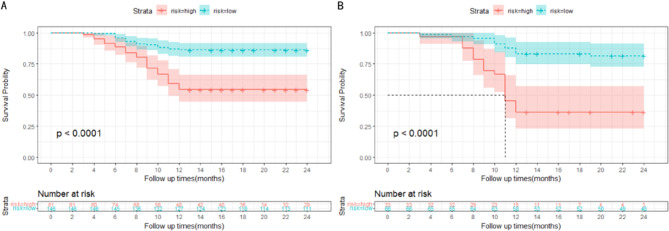
Kaplan-Meier curves of RFS for patients in low-risk and high-risk groups in training set (**A**) and validation set (**B**)

According to the multivariable Cox analysis, AFP, MVI, Edmondson-Steiner, surgical methods and surgical margin were integrated to build the nomogram of RFS (Fig. 7). ROC analysis of the nomogram revealed that AUC of 1-year and 2-year RFS respectively reached 0.839 (95%CI: 0.775–0.903) and 0.856 (95%CI: 0.806–0.905) in the training set, and 0.808 (95%CI: 0.719–0.896) and 0.874 (95%CI: 0.804–0.943) in the validation set (Fig. 8). The calibration curves of nomogram revealed a strong consistency between actual observation and prediction (Fig. 9). In addition, the nomogram demonstrated a significant positive net benefit from the risk of early recurrence, indicating its great clinical practical value in predicting RFS of SHCC (Fig. 10). The Kaplan-Meier survival analysis of training set and validation set showed a distinct difference in survival rate (Fig. 11, *P* < 0.001).

## Discussion

In this study, we developed and validated two nomograms based on readily available preoperative and postoperative clinical data, one for preoperative prediction of SHCC with MVI, and one for prediction of early recurrence risk of SHCC after radical resection. MVI is the main factor determining treatment strategies, so that preoperative prediction of SHCC with MVI can guide the selection of surgical methods, and prediction of postoperative early recurrence risk can also provide a basis for selection of postoperative adjuvant treatment plans, which is of great importance in prognosis improvement [26, 27]. By calculating AUC and plotting calibration curves, we have shown that both nomograms have good predictive performance and consistency, demonstrating a good predictive value, which was beneficial for preoperative non-invasive prediction of SHCC with MVI as well as the risk of early recurrence after radical resection, and provided a more accurate guidance for the intervention and treatment of SHCC patients. In addition, the DCA results indicated that the nomograms have good clinical application value and is beneficial for personalized treatment interventions.

MVI mainly refers to the nesting clusters of cancer cells seen microscopically in the endothelial cell-lined vascular lumen, which is the initial stage of portal vein cancerous embolism [28]. Postoperative pathology is still the gold standard for confirming the diagnosis of MVI. MVI mainly reflects the invasive nature of HCC, and it is an important predictor of postoperative recurrence of HCC. Shindoh et al [29] have demonstrated that even in SHCC, MVI is still an independent risk factor for poor prognosis, including increased risk of recurrence and decreased long-term survival. Therefore, preoperative prediction of MVI can not only guide the selection of surgical methods, but also provide a basis for the selection of new adjuvant plans before surgery, ultimately improving survival outcomes. As mentioned earlier, there are few studies on the occurrence of MVI in patients with SHCC. Zhang et al [30]found that fibrinogen, AFP, cirrhosis, tumor diameter and poor tumor border were independent risk factors of HCC with MVI, and similar to Zhang’s study, our study found that serum AFP level, tumor diameter and tumor margins were independent risk factors for SHCC patients with MVI.

Regarding tumor diameter, numerous studies have previously demonstrated that tumor size is an independent prognostic factor in HCC patients [31–33]. NLR, serving as an inflammatory indicator, has been reported to be associated with the poor prognosis of HCC [34, 35]. Interestingly, we found that tumor diameter and NLR were independent risk factors for MVI rather than independent prognostic factors for SHCC. The reason may be that the endpoint of our study is different from previous studies, cause our study only focused on predicting early recurrence. In addition, different study populations (our study only included SHCC) might be an another reason.

Edmondson-Steiner grade has been identified as an independent risk factor for HCC recurrence. Zhou et al [36] proved that the Edmondson-Steiner grade had important significance for the prognosis of HCC and might become a key prognostic indicator for HCC without MVI. Our study found that the early recurrence rate in Edmondson-Steiner III-IV stage patients was significantly higher than that of I-II, which confirmed this viewpoint. AFP is a specific tumor marker for HCC with a specificity of up to 93.3%for early diagnosis [37]. Relevant studies have proved that the higher the serum AFP level, the shorter the survival time of HCC patients, indicating the close relationship between AFP level and prognosis [38, 39]. The results of our study showed that serum AFP level was not only an independent risk factor for the occurrence of MVI, but also an independent risk factor for early recurrence of SHCC, which was consistent with previous research [40].

Surgical methods and surgical margin are another factor affecting HCC recurrence. Many studies have shown that AR has a better prognosis than NAR [41, 42], however, Eguchi et al [43] found that for SHCC, AR was not beneficial. Therefore, the therapeutic effect of AR remains controversial. Our results showed that AR improved patient prognosis and reduced early recurrence of HCC compared with NAR, the reason might be that AR could remove intrahepatic lesions and microvascular metastases. Famularo et al [44] found that the risk of early recurrence of HCC after AR was significantly reduced, especially in HCC with MVI. Therefore, if SHCC patients have sufficient liver function reserve and AR is technically feasible, AR should be considered first, and NAR should be considered as an alternative therapy for patients with limited liver function reserve [41]. In addition, Su et al [45] revealed that the RFS of wider surgical margin (≥ 1 cm) was higher than that of narrower surgical margin in HCC. Our results showed that wide resection margin (≥ 1 cm) can improve the prognosis of all patients, which is consistent with previous reported studies. Therefore, we suggested surgeons should use AR method as much as possible, and try to preserve the surgical resection margin width ≥ 1 cm for SHCC patients.

Our study has several limitations. First, this study was a single-center retrospective study with a limited sample size. Second, the cut-off values of some indicators in this study had a certain subjectivity, which might have a certain impact on the study results. Finally, most of the patients in this study suffered from hepatitis B virus-related hepatocellular carcinoma, which might have some selection bias. In the future, large-sample, multi-center prospective studies are planned to further improve and validate the results.

## Conclusion

Our study developed and validated a preoperative nomogram for MVI prediction, and a prognostic nomogram for early recurrence in SHCC patients. These nomograms could better predict individual survival, guide follow-up management strategies and provide a basis for clinical decision making. Furthermore, based on the prognostic nomogram, we suggested that surgeons should choose AR while trying to maintain a surgical margin of ≥ 1 cm, which could reduce early recurrence and improve the prognosis of SHCC patients.

## Data Availability

The data used and evaluated in this study are not openly available due to reasons of sensitivity and are available from the corresponding author upon reasonable request. The data are located in the controlled access data storage of Union Hospital of Tongji Medical College, Huazhong University of Science and Technology.

## Abbreviations

MVI: Microvascular invasion
SHCC: Small hepatocellular carcinoma
LH: Laparoscopic hepatectomy
RFA: Radiofrequency ablation
BCLC: Barcelona Clinic Liver Cancer
RFS: Recurrence-free survival
EOB-MRI: Gadoxetic acid–enhanced magnetic resonance imaging
AST: Aminotransferase
ALT: Alanine aminotransferase
TBIL: Total bilirubin
ALB: Albumin
PLT: Platelet
PT: Prothrombin time
AFP: Alpha-fetoprotein
PLR: Platelet-to-lymphocyte ratio
NLR: Neutrophil-to-lymphocyte ratio
SIRI: Systemic inflammation response index
SII: Systemic immune-inflammation index
ANRI: Aspartate aminotransferase to neutrophil ratio index
PNI: Prognostic nutritional index
AR: Anatomic resection
NAR: Non-anatomic resection
RFS: Recurrence-free survival
